# Immune-Based Strategies for Pancreatic Cancer in the Adjuvant Setting

**DOI:** 10.3390/cancers17071246

**Published:** 2025-04-07

**Authors:** Kai-Li Liang, Nilofer S. Azad

**Affiliations:** Department of Oncology, Sidney Kimmel Comprehensive Cancer, Johns Hopkins University School of Medicine, Baltimore, MD 21205, USA; kliang14@jhmi.edu

**Keywords:** pancreatic cancer, immunotherapy, adjuvant therapy, cancer vaccine, immune checkpoint inhibitor, clinical trial

## Abstract

Pancreatic ductal adenocarcinoma (PDAC) is the third leading cause of cancer-related mortality in the United States, with poor overall survival across all stages. Less than 20% of patients are eligible for curative surgical resection at diagnosis, and despite adjuvant chemotherapy, most will experience disease recurrence within two years. The incorporation of immune-based strategies in the adjuvant setting remains an area of intense investigation with unrealized promise. It offers the potential of providing durable disease control for micro-metastatic disease following curative intent surgery and enabling personalized treatments based on mutational neoantigen profiles derived from resected specimens. However, most of these attempts have failed to demonstrate significant clinical success, likely due to the immunosuppressive tumor microenvironment (TME) and individual genetic heterogeneity. Despite these challenges, immune-based strategies, such as therapeutic vaccines targeted towards neoantigens, have demonstrated promise via immune activation and induction of T-cell tumor infiltration. In this review, we will highlight the foundational lessons learned from previous clinical trials of adjuvant immunotherapy, discussing the knowledge gained from analyses of trials with disappointing results. In addition, we will discuss how these data have been incorporated to design new agents and study concepts that are proving to be exciting in more recent trials, such as shared antigen vaccines and combination therapy with immune-checkpoint inhibitors and chemotherapy. This review will evaluate novel approaches in ongoing and future clinical studies and provide insight into how these immune-based strategies might evolve to address the unique challenges for treatment of PDAC in the adjuvant setting.

## Simple Summary

Pancreatic cancer is the third most common cause of cancer-related death in the United States. Despite curative intent surgical resection and adjuvant chemotherapy, most patients will eventually have disease relapse, highlighting the unmet need for improved treatments in the adjuvant setting. In multiple other histologies, adjuvant immunotherapy has emerged as an effective approach in eliminating residual disease. While previous efforts to incorporate immunotherapy into the treatment of pancreatic cancer, including therapeutic cancer vaccines, immune checkpoint inhibitors, and cell-based therapies, have had limited clinical success, recent developments have reinvigorated the field. In this review, we discuss valuable lessons learned from prior clinical trials that have shaped our understanding of the pancreatic tumor immune environment and foundational insights that have paved the way for innovative approaches for treating pancreatic cancer with immune-based strategies in the adjuvant setting.

## 1. Introduction

### 1.1. Unmet Need for Improved Adjuvant Treatments for Pancreatic Cancer

Pancreatic ductal adenocarcinoma (PDAC) is the third most common cause of cancer-related mortality in the U.S., with a dismal 5-year overall survival (OS) rate of <10% [1,2]. The majority of patients with PDAC are diagnosed with unresectable or metastatic disease (80–85%), while only approximately 15–20% have surgically resectable disease [1]. Despite curative-intent surgical resection, recurrence rates remain high, at approximately 60% within 3 years of surgical resection, likely owing to preexisting micro-metastatic disease and microscopically undetectable residual disease within the tumoral bed [3,4,5,6].

After the widespread adoption of adjuvant gemcitabine following the CONKO-001 trial, disease-free survival (DFS) increased nearly two-fold, bringing the five-year overall survival to approximately twenty percent in patients receiving adjuvant gemcitabine compared to observation [7]. Following the ESPAC-4 trial, which demonstrated improved overall survival with combination adjuvant gemcitabine and capecitabine (Gem/Cap) compared to gemcitabine monotherapy, Gem/Cap became the new standard of care (SOC) for PDAC patients following resection [6]. In the subsequent PRODIGE 24-ACCORD trial, patients receiving modified folinic acid, fluorouracil, irinotecan, and oxaliplatin (mFOLFIRINOX) outperformed single-agent gemcitabine, which led to approval of mFOLFIRINOX for physically fit patients following definitive surgery [5]. Despite the adoption of adjuvant mFOLFIRINOX and Gem/Cap, multiagent chemotherapy remains highly unspecific and does not prevent disease recurrence; it has a median DFS of only 16.6–21.6 months and median OS of 40.5–54 months, highlighting the unmet need for more effective adjuvant therapies for PDAC [5,8].

### 1.2. Obstacles for Adjuvant Immunotherapy

The use of immunotherapy, specifically immune checkpoint inhibitors (ICI), in the adjuvant setting has yielded significant improvements in patient outcomes for multiple solid tumor types. After showing initial success in metastatic patients, ICI therapy has demonstrated a substantial survival benefit and is now the standard of care in the adjuvant setting in multiple tumor types, including lung cancer, melanoma, urothelial cancer, and others [9,10,11,12,13]. Unfortunately, this success has not translated for the majority of PDAC patients. Patients with advanced and metastatic PDAC have failed to respond to single agent or combination ICIs, with zero patients having an objective response to anti-PD-L1 or anti-CTLA-4 monotherapy and only a 3.1% objective response rate (ORR) with combination therapy [14,15,16,17].

Explanations for PDAC’s insensitivity to immunotherapy or immunologically ‘cold’ phenotype have been attributed to many features, including a low tumor mutational burden (TMB) with resultant lower expression of neoantigens, which fail to attract T cells into the tumor microenvironment (TME) [18,19]. Furthermore, the immunosuppressive environment in PDAC presents with barriers for immune infiltration, including the dense extracellular matrix composed of collagen, glycosaminoglycans and proteoglycans produced by stromal cells, stellate cells, and cancer-associated fibroblasts (CAFs) leading to a highly fibrotic environment [20,21]. The PDAC TME is also enriched for populations of regulatory T cells (Tregs), which suppress anticancer immunity, myeloid derived suppressor cells (MDSCs) or immature myeloid cells that suppress T-cell responses, and M2 tumor-associated macrophages (TAMs), which promote tumor growth and metastasis [22,23,24]. Collectively, these immunosuppressive populations are associated with shorter patient survival [25,26].

In addition to the inherently immunosuppressive TME, immune changes that occur post-surgically are challenges that must be overcome. While surgery is associated with a transient increase in total circulating white blood cells, the number of circulating T lymphocytes decreases, specifically creating an imbalance between immunosuppressive Tregs versus immune promoting helper T cells and cytotoxic T cells [27]. This immunosuppressive effect has been observed for weeks following surgery [28].

### 1.3. Opportunities for Adjuvant Immunotherapy

Despite these numerous obstacles with immunotherapy in the advanced and metastatic setting, incorporation of immunotherapy for PDAC in the adjuvant setting possesses several unique opportunities.

Perhaps the most compelling advantage of immunotherapy in the adjuvant setting is its relationship to the level of disease burden. Following definitive resection, adjuvant immunotherapy may be effective when the disease is limited to micro-metastatic disease, rather than bulky densely fibrotic tumors seen in more advanced stages, allowing T cells to target and eliminate residual disease [29]. In contrast to cytotoxic chemotherapy, whose anti-tumor effects last only during the period of administration, immunotherapy can activate an ongoing immune response, which in turn leads to tumor identification and elimination. This process of engaging the immune system can often take weeks or months to commence; however, responses can often be durable, even years after cessation of therapy [30]. Historically, many immunotherapy approaches in PDAC have focused on the advanced and metastatic setting; however, given that the median overall survival in metastatic pancreatic cancer patients, despite chemotherapy, is 8–11 months, the slower kinetics of vaccination and immunotherapy may prevent observable clinical benefit [31,32]. As such, earlier adoption of immunotherapeutic agents, particularly in the adjuvant setting, may be strategically suited to provide benefit even if only providing modest decreases in growth rate. Furthermore, with the increasing validation and incorporation of commercially available circulating tumor DNA (ctDNA) assays, adjuvant immunotherapy may be poised to intervene in the minimal residual disease (MRD) positive setting, where disease burden is low and where there is presently no standard of care treatment. On a molecular level, earlier intervention may be able to preempt the loss of human leukocyte antigen (HLA) class I molecules observed in more advanced disease, thus allowing for preservation of HLA class I molecules in their function to present peptides to T cells [33].

With adjuvant chemotherapy now considered as the standard of care in PDAC, immunotherapy may be well-positioned to leverage the mechanistic effects of chemotherapy. Overcoming the immunosuppressive tumor microenvironment remains one of the main barriers for immunotherapy success in PDAC. Exposure to cytotoxic chemotherapy agents such as cyclophosphamide, oxaliplatin, and gemcitabine have been shown to enhance antigenicity and immunogenicity of tumors by promoting adaptive immune responses [34]. Historically, cyclophosphamide (Cy) has been incorporated to suppress immunosuppressive cells, such as Tregs, to remodel the TME for subsequent immune-based therapy [35]. Notably different routes of administration, timing, and dosing of Cy have been shown to impact not only Treg number, but also functionality [36]. Additionally, gemcitabine has also been shown to reduce Tregs and MDSCs in patients with PDAC, suggesting that pretreatment with gemcitabine may condition the TME for subsequent immunotherapy [37].

Finally, although PDAC lacks the neoantigen diversity seen in other tumors such as melanoma, recent studies have demonstrated that nearly all PDAC cases express some candidate neoantigens, albeit with a heterogeneous distribution, with predicted neoantigens varying from four to four-thousand in some analyses [38]. Given this genetic heterogeneity seen in PDAC, genomic profiling from resected specimens provides an opportunity for defining novel immune-subtypes of PDAC that may aid in personalizing and predicting sensitivity to immunotherapeutic strategies. Availability of complete tumor specimens, obtained surgically, may provide more comprehensive tumor profiles and neoantigen identification, thus circumventing challenges associated with inadequate tissue often encountered from diagnostic biopsy specimens.

**Figure 1:**
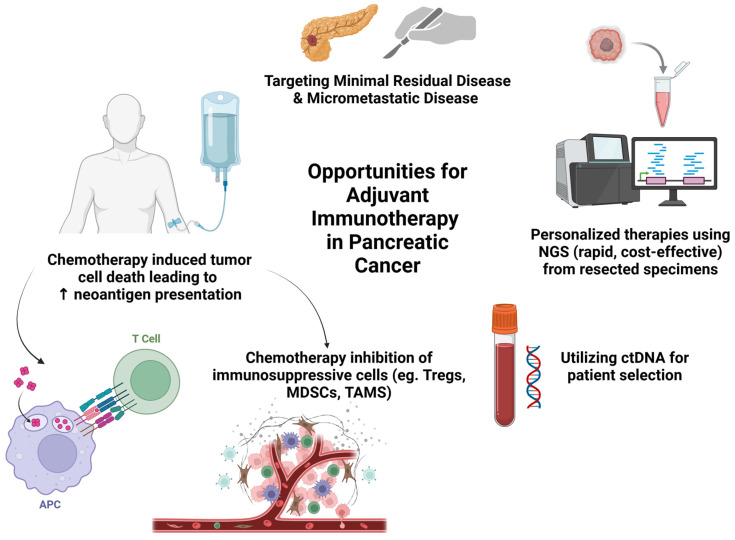
Opportunities for Adjuvant Immune-based Strategies in Pancreatic Cancer. Created in BioRender. https://BioRender.com/u41g223 (accessed on 4 February 2025).

Collectively, these opportunities for adjuvant immunotherapy in pancreatic cancer have formed the rationale for historical and ongoing clinical trials [Figure 1].

In this review, we will examine the historical and current landscape of clinical trials utilizing immune-based strategies in the adjuvant setting for pancreatic cancer. These adjuvant immune-based approaches have primarily been in the context of (1) cancer vaccines and (2) adoptive cellular therapies. Although multiple immune-based approaches have been examined in the advanced and metastatic setting, this review will primarily focus on clinical approaches in the adjuvant setting. Selected completed and ongoing clinical trials involving immunotherapy for pancreatic cancer in the adjuvant setting are summarized in Table 1.

## 2. Vaccine Therapies

### 2.1. Rationale for Vaccine-Based Immunotherapy

In the simplest terms, cancer cells are the result of genetic and epigenetic alterations that fundamentally change their composition and function from native healthy cells. As a result, various antigens are expressed, some exclusively expressed in tumor cells, known as neoantigens, and others that are concomitantly expressed in healthy cells but are merely over-expressed in tumor cells, known as tumor associated antigens (TAAs). Following antigen recognition by professional antigen presenting cells (APCs) or dendritic cells (DCs), major histocompatibility complex (MHC) proteins, both class I and II, present these processed antigens to activate tumor-specific CD8+ and CD4+ T cells, ultimately leading to tumor cell death. Therapeutic cancer vaccines aim to exploit T cell activation and destruction of cancer cells. Over the past 30 years, multiple groups have employed various principles of this mechanism, which has led to the creation of a diverse collection of cancer vaccines.

### 2.2. Whole Cancer Cell Vaccines

#### 2.2.1. GVAX Vaccine

In the late 1990s, the allogeneic granulocyte-macrophage colony-stimulating factor (GM-CSF)-secreting tumor vaccine or GVAX vaccine emerged as the first whole cancer cell vaccine. Whole cancer cell-based vaccines ideally provide the optimal template for generating antitumor immunity due to their display of a wide variety of possible TAAs. In the phase I study conducted by Jaffee et al., 14 patients with Stage I-III PDAC were vaccinated with GVAX vaccine following surgical resection [39]. Following vaccination, patients received 6 months of adjuvant radiation and chemotherapy. Of those vaccinated, 3/14 developed a delayed type hypersensitivity (DTH) response to autologous tumor cells, which correlated with increased disease-free survival time and these subjects remained disease-free for at least 25 months following diagnosis [39]. Encouraged by these results, a subsequent phase II study was conducted with 60 patients with stage I or II PDAC receiving GVAX vaccine for 8–10 weeks following resection, followed by adjuvant 5-FU based chemoradiation [40]. The major findings of this trial demonstrated that this sequential combination of GVAX vaccine plus chemoradiotherapy was safe and well tolerated, with a median DFS of 17.3 months. Strikingly, induction of mesothelin-specific CD8+ T cells in HLA-A1+ and HLA-2+ patients correlated with disease free survival [40]. Conclusions from these trials revealed that this combination of GVAX followed by chemoradiation was well tolerated and compared favorably with the existing historical OS rates in resected cancer (15–20 months).

Lessons from these early GVAX trials also highlighted the need to address infiltrating Treg populations contributing to the immunosuppressive TME, prompting a subsequent neoadjuvant/adjuvant trial of GVAX in combination with low dose Cy [41]. While Cy was shown to reduce Tregs in tumor specimens as intended, this did not translate into clinical outcomes, although not powered to compare survival. Arguably, however, the most compelling insights gained from this trial were that during the histological analysis of resected specimens, 33/39 patients developed vaccine-induced intratumoral tertiary lymphoid aggregates (TLAs). These TLAs resembled naturally induced lymph node-like structures previously seen in other more immunogenic cancers, such as melanoma and non-small cell lung cancer (NSCLC), in which TLAs were associated with response to ICI and improved survival [61,62]. Indeed, analyses of these specimens demonstrated that decreased Tregs within the vaccine-induced TLAs was associated with increased intratumoral Teffector/Treg ratios and improved patient survival. This study marked the first example of a cancer vaccine converting a “non-immunogenic” tumor into an “immunogenic” neoplasm via T-cell infiltration and development of TLAs [41,42]. Lastly, another critical insight gained was that following GVAX administration, T cell infiltration and the upregulation of immunosuppressive pathways including the PD-1/PD-L1 were observed. This observation provided a potential explanation for the historical failures of GVAX monotherapy and provided key evidence that vaccination may prime the TME for subsequent exposure to immune checkpoint inhibition.

Building on the insights, a platform neoadjuvant/adjuvant study was designed to evaluate potential synergy with anti-PD-L1 inhibition [43]. This study of 40 patients contained 3 arms: GVAX with low dose Cy (Arm A), GVAX with the anti-PD-1 antibody nivolumab (Arm B), and GVAX vaccine with both nivolumab and urelumab, an agonist of the T-cell costimulatory molecule CD137 (Arm C) [43]. Two weeks prior to surgical resection, patients received a single priming treatment per their respective arm, and 6–10 weeks following definitive surgical resection, patients resumed their assigned therapy along with SOC adjuvant chemotherapy. The triplet arm of GVAX, nivolumab, and urelumab reached its primary endpoint of increasing intratumoral CD8+CD137+ cells, with the median DFS more than doubled for the three-drug regimen (33.5 months) compared to GVAX and nivolumab (15.0 months) [43]. OS also favored the triplet regimen compared to the doublet but was underpowered to reach statistical significance (35.6 months vs. 27.0 months). These results ultimately helped form the foundational hypothesis that combining vaccines and ICIs may lead to robust and durable tumor immune responses via synergistic or complimentary mechanisms.

The optimal sequence of GVAX and conventional therapies including radiation has remained controversial. Factors related to lymphopenia induced by standard chemoradiation have raised concerns about compromising vaccine efficacy and immune activation. Conversely, in the earliest NSCLC immunotherapy trials with pembrolizumab, the subgroup of patients who received radiotherapy prior to immunotherapy in fact had significantly improved PFS and OS, suggesting an immune priming effect of radiotherapy [63]. To explore this question, stereotactic body radiation therapy (SBRT) was proposed as it has been demonstrated to result in less lymphopenia and potentially less immunosuppression when compared to standard chemoradiation [64]. In 19 patients with high-risk resected PDAC (i.e., close/positive margins within ≤1 mm and/or lymph node metastasis), SBRT was evaluated in combination with mFOLFIRINOX + Cy/GVAX [44]. In the SBRT + mFOLFIRINOX + Cy/GVAX cohort, DFS was 24.1 months with a compelling median OS of 61.3 months, which numerically was superior to the overall cohort (DFS 18.2 months; median OS 36.2 months). Results from this study, while limited by sample size, underscore the potential of multimodal combination of SBRT, cancer vaccine, and chemotherapy, especially for high-risk patients who require adjuvant therapy.

In summary, lessons learned from the GVAX trials have repeatedly shown that while vaccination is able to generate immune activation and the formation of TLAs, vaccination alone is unable to overcome immune suppressive populations within the TME (e.g., Tregs) and the subsequent upregulation of immune checkpoints (e.g., PD-1). Strategies sequencing lymphodepleting therapy, including Cy, to precondition the TME, followed by ICIs may be more effective in leading to robust and durable responses. GVAX in combination with anti-PD-1 therapy and a CD137 agonist is currently being examined in the neoadjuvant/adjuvant setting (NCT06782932).

#### 2.2.2. Algenpantucel-L

Algenpantucel-L (hyperacute-pancreatic cancer vaccine, HAPa) consists of genetically modified, irradiated, live, human allogeneic tumor cells that express murine α-1,3-galactosyltransferase, an enzyme involved in the synthesis of α-1,3-galactosylated epitopes on cell surface proteins [65]. HAPa exploits the hyperacute rejection mechanism in xenotransplantation to target pancreatic cancer cells [45]. In their multi-institutional, open-label, phase II trial, Hardacre et al. evaluated the addition of algenpantucel-L to SOC adjuvant gemcitabine-based chemoradiotherapy for 70 patients following resection. With a median follow up of 21 months, the 1-year disease-free survival was 62% and 1-year overall survival was 86% improved from the historical control at that time. Unfortunately, despite the early success in the phase II trial, data from the phase III trial of neoadjuvant chemotherapy with or without Algenpantucel-L in borderline or locally advanced unresectable PDAC failed to improve overall or progression-free survival, ceasing clinical development of this approach [65].

The authors have suggested that a potential explanation for the lack of improved survival may have been more attributed to the lower surgical resection rate, which was driven by the lower resectability status at baseline. In this study, the vast majority (82%) of patients were locally advanced unresectable, whereas most other historical studies included more equally distributed borderline resectable and locally advanced PDAC patients. Furthermore, correlative immunologic data were not generated, thus limiting conclusions to explain the biological basis for the lack of clinical benefit. Extrapolating from other contemporary vaccine studies, the lack of combinatorial immunotherapeutic agents to address the TME and upregulation of checkpoints likely contributed to its failure.

### 2.3. Peptide Vaccines

#### 2.3.1. KRAS Targeting Peptide Vaccines

Expressed in up to 90% of all patients with PDAC, the mutated oncoprotein KRAS (Kirsten rat sarcoma virus) is an attractive neoantigen vaccine target that can be harnessed immunologically for vaccination [66]. The most common *KRAS* oncogene mutations occur in codons 12, 13, and 61 and their presence has been well-documented to lead to increased tumor proliferation and worse overall survival [67]. The mutant KRAS (mKRAS) signaling pathway also contributes to the immunosuppressive TME through upregulation of PD-L1 expression, downregulation of MHC class I, and the secretion of cytokines, which transform CD4+ T cells into immunosuppressive Tregs [68,69,70]. In contrast to TAAs, mKRAS is a neoantigen that arises during tumorigenesis and thus is exempt from central tolerance and poised to elicit effective T-cell responses [71].

The earliest KRAS peptide-based vaccine trial of pancreatic patients was the Phase I/II study by Gjertsen et al., where a synthetic mutant RAS peptide vaccine containing residues 5–21 of p21 RAS was given in combination with GM-CSF to 48 patients with PDAC (10 surgically resected, and 38 with advanced disease) [46]. For resectable patients, a single mutant RAS peptide corresponding to the patient’s KRAS mutation was given, whereas patients with non-resectable disease were given a mixture of the four most frequent mutant KRAS peptides. In 58% of evaluable patients (n = 25/43), peptide-specific immunity was induced and, among responders, the median survival was 148 days vs. 61 days in non-responders (*p* = 0.0002) [46]. In another study of resected pancreatic cancer patients with codon 12 KRAS mutation, 21-mer peptide vaccines corresponding to the patient’s codon 12 mutation were administered with the GM-CSF to 24 patients monthly for 3 months [47]. The median recurrence free survival (RFS) was 8.6 months and overall survival was 20.3 months. One patient had a detectable immune response specific to the their KRAS mutation and three patients displayed a non-specific DTH; however, in contrast to the mutant RAS peptide vaccine reported by Gjertsen et al., there was no relationship between immune response and clinical outcome. Lessons from these studies emphasized the requirement for increasing vaccine immunogenicity and utilizing multivalent and pooled peptide vaccines.

#### 2.3.2. Targovax TG01

Targovax TG01 is a pooled peptide vaccine of seven RAS peptides of the most common codon 12 and 13 mutations in KRAS. In the multicenter phase I/II study, 32 patients received TG01 plus GM-CSF with adjuvant SOC gemcitabine following surgical resection [48]. In the initial 19 patients treated, 4 serious adverse reactions were observed and were considered to be attributable to TG01 treatment. As a result, a modified vaccination schedule, with no vaccinations during gemcitabine chemotherapy, was implemented with no serious adverse reactions. In the main cohort of 19 patients, 95% had an immune response compared to 92% in the modified cohort. Survival for the main cohort vs. the modified cohort were as follows: median overall survival (33.1 months vs. 33.4 months) and median disease-free survival (13.9 months vs. 19.5 months) [48].

The preliminary success of this regimen has provided the rationale for the current phase II trial of TG01 RAS vaccine plus QS-21 (Stimulon^®^, SaponiQx, Lexington, MA, USA) adjuvant with or without balstilimab (AGEN2034; anti-PD-1) in MRD positive patients following surgical resection and SOC adjuvant chemotherapy (NCT05638698) [49]. MRD will be detected via commercially available ctDNA assays (Signatera, Natera, Austin, TX, USA) with somatic variants detected through whole-exome sequencing of primary tumor and matched patient blood samples. Six-month disease control rate (ctDNA stable, decreased, or cleared) is the primary objective. Unique features of this trial include employing combination vaccine and immune checkpoint inhibitor in the MRD setting and the incorporation of anti-PD-1 balstilimab [72].

#### 2.3.3. GI-4000

The GI-4000 product is a yeast-based product targeting the seven most common *ras* mutations at codons 12 and 61. This technology was developed using heat-killed recombinant *Saccharomyces cerevisiae* yeast as a vector, engineered to express target protein antigens, with the goal of activating dendritic cells and generating T cell cytotoxicity towards cancer cells. In a randomized phase II trial, adjuvant gemcitabine vs. gemcitabine plus GI-4000 was evaluated in patients with resected pancreatic cancer [50]. Patients (n = 176) received 10^7^ yeast cells of GI-4000 containing only the mutation identified in his or her tumor for three weekly doses starting 21 to 25 days after resection. The primary endpoint was an RFS that was not statistically different between the GI-4000 and placebo groups. Disappointingly, for R0 resection subjects, no increases in IFNγ-ELISpot responses were observed for those who received GI-4000; however, a reduction in Tregs was observed in R0/R1 subjects treated with GI-4000 compared to placebo (*p* = 0.033). Several clinical trials subsequently employed the GI-4000 vaccine in combination with chemotherapy, ICI, anti-VEGF, and adoptive T cell transfer in both the adjuvant and advanced/metastatic setting; however, these trials have been terminated due to low enrollment or withdrawn.

#### 2.3.4. ELI-002

In the phase I AMPLIFY-201 trial, the cancer vaccine ELI-002 2P, utilizing amphiphile modification of two mKRAS peptides, G12D and G12R (Amph-Peptides-2P), together with CpG oligonucleotide adjuvant (Amph-CpG-7909) were given to 20 patients with PDAC and 5 patients with colorectal cancer who were positive for MRD (ctDNA) and/or persistently elevated serum tumor antigens (CA 19-9 and CEA) after locoregional treatment [51]. In 21/25 patients, direct ex vivo mKRAS-specific T cell responses were observed (59% both CD4+ and CD8+ T cells). Tumor biomarker decreases from baseline were seen in 21/25 patients and complete ctDNA clearance (0 mean tumor molecules per milliliter (MTM/mL) was observed in 6/25 patients, which included 3 PDAC patients [51].

Several features of this trial are worth highlighting, which provide insights into future vaccine and trial design. Engineered with both HLA class I and II epitopes, the ELI-002 2P mKRAS Amph-peptides were observed to stimulate both CD4+ and CD8+ T cell responses. This dual CD4+ and CD8+ T cell response supports not only the anti-tumor potential of CD8+ T cells, but also the CD4+ T cell support of CD8+ T cell activation and direct cytotoxic activity [51]. One insightful observation into clinical application was the association of RFS and baseline absolute lymphocyte count, suggesting that effective vaccine response may be dependent on hematologic recovery following cytotoxic chemotherapy. Taken together, this information may aid in predicting the optimal timing of sequential therapy. Lastly, this trial was designed with a specific focus on utilizing tumor-informed ctDNA to enroll patients with positive MRD and using serum biomarker reduction and ctDNA clearance as a marker for vaccine efficacy. The phase II randomized trial (AMPLIFY-7 trial) is currently active and evaluates the safety and efficacy of ELI-002 7P (7-peptide formulation) as adjuvant monotherapy in patients with mKRAS PDAC following surgical resection and chemotherapy with or without radiation (NCT05726864).

#### 2.3.5. Pooled KRAS Peptide Vaccine

In an ongoing pilot study of 12 patients with resected PDAC (NCT04117087), a pooled KRAS vaccine combined with ipilimumab and nivolumab was demonstrated to be safe and well tolerated [52,73]. This pooled KRAS synthetic long peptide (SLP) vaccine targets six common KRAS G12 and G13 mutations and is coupled with the immune adjuvant poly-ICLC (polyinosinic-polycytidylic acid (poly-IC) that is stabilized with poly-l-lysine (PLL) and carboxymethylcellulose). Poly-ICLC is a synthetic RNA toll-like receptor 3 (TLR3) agonist that stimulates innate immunity similar to antiviral immunity and has a clinical record for inducing T cells when given with peptide vaccines [74,75]. Preliminary data demonstrated that this pooled mutant KRAS peptide vaccine induced robust de novo mKRAS-specific T cells in peripheral blood, and patients (n = 8/11) who mounted a >5–fold increase in IFNγ-producing mKRAS-specific T cells within 17 weeks post-vaccination displayed improved disease free survival compared to non-responders (not reached vs. 2.8 months; *p* = 0.045) [52].

One distinction of this study is that it is the first vaccine study in the adjuvant PDAC setting that has employed combination dual ICI therapy (PD-L1 and CTLA-4). Future studies with larger sample sizes will be needed to validate this dual checkpoint inhibition approach in the adjuvant setting, though trials with this vaccine and dual ICI therapy are ongoing in the first line metastatic maintenance setting (NCT06411691).

#### 2.3.6. Personalized Neoantigen Peptide Vaccines

Similar to shared neoantigens such as mutant KRAS, personalized neoantigens are selectively expressed on tumor cells and have strong affinity for MHC, thus evading central tolerance and mitigating the toxicity caused by the lack of specificity of TAAs [76]. Personalized neoantigens, however, have distinct mutations that are uniquely identified from the genetic profile of each patient. In the adjuvant setting, access to resected specimens allows for sufficient tissue for genome sequencing and ideally maximizes capturing the vast array of mutations that may not be identified on limited biopsy samples due to tumor regional genetic heterogeneity.

In a single-center, phase 1b clinical trial (NCT03558945), 16 patients, following radical pancreatic resection and post-operative chemotherapy, received personalized neoantigen vaccines weekly for 1 month (priming phase) followed by 2 additional doses in weeks 12 and 20 (boosting phase). Preliminary data from vaccinated patients demonstrated a 3-year RFS of 56% and a 3-year OS of 74%, an improvement from historical controls with chemotherapy alone. Observations from single-cell sequencing revealed expansion of cytotoxic CD8+ T cells at the priming phase with CD4+ T cells during the boosting phase [53]. Additionally, the group reported a helper B-cell subtype that interacted with T cells in patients associated with prognosis, suggesting a potential role for B-cell epitopes in vaccine efficacy. Additional neoantigen peptide vaccine trials are being tested utilizing various adjuvants including GM-CSF (NCT04810910) and in combination with chemotherapy and immune-checkpoint inhibitor therapy (NCT06344156).

#### 2.3.7. HSP-Peptide Complex Vaccines

Heat shock proteins (HSPs) are an attractive vaccine target as they exist as carriers or chaperones of tumor antigens for presentation to APCs. The autologous heat shock protein family member HSPPC-96 (Oncophage^®^) was initially identified in rodents, and in animal models, HSPPC-96 isolated from individual tumors was shown to induce anti-tumor responses. In a phase I clinical trial, HSPPC-96 was administered weekly, for 4 weeks, to 10 patients with resected PDAC who neither received adjuvant radiation or chemotherapy [54]. Median overall survival was 2.2 years and autologous anti-HSPPC-96 ELISPOT reactivity increased significantly in 1/5 patients examined; however, there was no observed correlation between immune response and prognosis [54].

While this study demonstrated that isolating HSPPC-96 peptide complexes from resected specimens was feasible, it had disappointing clinical responses and was unable to elicit tumor immunity against unrelated tumors. Additionally, large scale production of such an autologous complex would pose logistical and technical challenges, limiting its widespread adoption.

### 2.4. Dendritic Cell Vaccines

Dendritic cells (DCs) are regarded as arguably the most powerful antigen-presenting cells (APCs) due to their exceptional ability to activate naïve T cells, leading to the formation of memory T and B cells that facilitate strong, antigen-specific immune responses. The scarcity of DCs in PDAC results in impaired immune surveillance; however, increasing DC populations in early PDAC lesions can rejuvenate the anti-tumor T-cell immune response [77]. Various research teams have sought to exploit these properties by isolating DCs, loading them with TAAs or mRNA encoding TAAs, and then reintroducing them into patients as vaccines. The first study to evaluate this approach included 12 patients with resected pancreatic (n = 10) and biliary tract tumors (n = 2), who received a mucin-1 (MUC1) peptide-loaded DC vaccine. MUC1 is a large membrane glycoprotein that is heavily glycosylated and restricted to the apical surface of epithelial cells; however, MUC1 in tumor cells is under-glycosylated and no longer restricted to the apical surface [55]. This change in expression leads to increased protein presentation to the immune system. Peripheral blood was collected from patients for generation of DCs and, following maturation, DCs were pulsed ex vivo with MUC1 peptide [55]. Of the 12 patients vaccinated, 4 patients (3 pancreatic) were alive and without disease recurrence 4 years following initial vaccination. Groups utilizing a DC vaccine platform have also started to employ targeting mutant KRAS G12 mutations. In a small phase I study, nine patients were vaccinated with autologous dendritic cells pulsed with one or more distinct short (nonamer or decamer) mutant KRAS peptides targeting patient-specific HLA I alleles [59]. Patients received a total of two vaccine doses (prime and boost) and no patients experienced any grade 3 or higher adverse events. Six of the nine vaccinated patients (67%) generated mutant KRAS specific T cell responses, and at median time of follow up of 25.3 months, five patients were without evidence of tumor recurrence [59].

In another study, by Lau et al., allogeneic-tumor lysate-based DC therapy (MesoPher) was administered in a phase I study of 10 patients. The allogenic tumor lysate is an ‘off-the-shelf’ product composed of several TAAs, generated from malignant mesothelioma cell lines, containing shared antigens with PDAC, including mesothelin (MSLN), WT1, and Survivin [56]. Following surgical resection and SOC adjuvant chemotherapy, patients received MesoPher at week 0, 2, and 4 and months 3 and 6. With a median follow up of 25 months, 7/10 patients did not experience disease recurrence or progression [56]. Flow cytometry of peripheral blood was collected following vaccination and patients demonstrated increased frequencies of memory CD4+ T cells expressing Ki67+ and PD-1+, a phenotype that, in NSCLC, is associated with clinical benefit after PD-1 ICI therapy [78]. MesoPher in combination with the CD40 agonistic antibody is ongoing in the metastatic setting to evaluate safety and treatment-induced tumor-specific immunological responses [57].

Ongoing studies into DC vaccines include the single center, single arm, Phase Ib trial evaluating subcutaneous DC vaccine loaded with personalized peptides (PEP-DC vaccine) in combination with SOC adjuvant chemotherapy including either mFOLFIRINOX (subgroup 1) or gemcitabine and capecitabine (subgroup 2), followed by nivolumab (NCT04627246) [58]. The DECIST trial is another ongoing phase I dose-escalation study for DOC1021, a Th-1 dendritic cell immunotherapy in combination with SOC chemotherapy for adjuvant PDAC treatment (NCT04157127). Primary endpoints include maximum tolerated dose and dose-limiting toxicities, with secondary endpoints including time to recurrence and OS. In July 2024, DOC1021 was granted FDA fast track designation for PDAC [79].

### 2.5. mRNA Vaccines

Following the success of messenger RNA (mRNA) vaccines for SARS-CoV-2 during the COVID-19 pandemic, mRNA vaccines have gained significant attention as a potential platform for cancer vaccine delivery [80,81].

In the phase I clinical trial by Rojas et al., 16 patients were treated sequentially with one dose of atezolizumab and autogene cevumeran, an individualized mRNA neoantigen vaccine containing up to 20 MHC class I and MHC class II restricted neoantigens synthesized in real time from resected PDAC tumors [60]. Atezolizumab (anti-PD-L1) was administered six weeks after resection and seven priming vaccine doses were given starting at week nine post-resection. Following this priming phase, at 21 weeks post-surgery, patients received 12 cycles of mFOLFIRINOX and a booster vaccine at week 46. Half of the patients (n = 8/16) mounted neoantigen-specific T cell responses against at least one vaccine neoantigen as measured by ex vivo INFγ ELISpot analysis. At median follow up time of 18 months, the responders had a median RFS that was not reached compared with the 8 non-responders who had a median RFS of 13.4 months (*p*  =  0.003, hazard ratio (HR)  =  0.08 (95% confidence interval (CI) 0.01–0.4). Amongst the 8 responders, vaccination expanded multiple clones (median 7.5 clones) from undetectable levels to up to 10% (median 2.8%). Interestingly, tumors of responders were observed to be more clonal, which the investigators postulated may represent immune-edited evolution, as seen in immunogenic PDAC long term survivors [60]. The authors also speculate on the possibility that a more clonal primary tumor may be more recognizable to the host immune system, thus promoting greater response to vaccination.

Advantages of an mRNA approach include both safety and modifiability with which individualized antigens can be manufactured as an mRNA payload. In contrast to other vaccine platforms that require immune adjuvants to increase immunogenicity, mRNA is intrinsically immunogenic, functioning as a pathogen-associated molecular pattern with affinity for multiple toll-like receptors (e.g., TLR7) [82,83]. The phase II, open label, multicenter study of this combinatorial based strategy with autogene cevumeran plus atezolizumab and mFOLFIRINOX versus mFOLFIRINOX alone in resected PDAC patients is currently ongoing (NCT05968326). Several additional phase I clinical trials are currently evaluating personalized mRNA neoantigen vaccines in combination with either anti-PD-1 (NCT06496373) or anti-CTLA-4 and chemotherapy (NCT06353646) in the adjuvant setting.

## 3. Adoptive Cellular Therapies

### 3.1. CAR T Cell Therapy

T cell activation and infiltration into tumor tissue has demonstrated deep and durable responses throughout solid tumors. As such, genetically engineering T cells for therapeutic use is being widely investigated. Chimeric antigen receptor (CAR) T cells, developed in the 1980s, have seen tremendous success in hematologic malignancies, with B-cell acute lymphocytic leukemia (ALL) patients exhibiting 80–90% complete remission rates in relapsed and refractory disease [84]. While native T cells require MHC antigen presentation, CAR T cells are able to bind directly to cancer cell surface proteins, glycolipids, and carbohydrates [85]. Within PDAC, various target antigens currently being investigated include MUC1, MSLN, carcinoembryonic antigen (CEA), prostate stem cell antigen (PSCA), and human epidermal growth factor receptor 2 (HER2) [86]. Additional proposed targets include targeting the stromal tissue and extracellular matrix, targets which have proved resistant to chemotherapy and which have thwarted effective T cell infiltration. A multi-center, phase Ib clinical trial is evaluating CT041, an autologous CAR T cell therapy targeting Claudin 18.2 (CLDN18.2) in subjects with CLDN18.2 expression-positive pancreatic cancer following adjuvant chemotherapy (NCT05911217). CLDN18.2 is a tight junction protein that is highly expressed in patients with PDAC (upwards of 59.2%) and has been associated with initiation, progression, and metastatic potential in PDAC [87]. CD041 has been studied in the metastatic setting for pancreatic cancer, demonstrating an overall response rate of 16.7% and disease control rate of 70.8% in previously treated PDAC in a small study [88]. In this upcoming adjuvant trial, patients will receive up to three treatments of CT041 autologous CAR T cell infusion. The primary objective is DFS with secondary objectives including safety, metastasis free survival, OS, pharmacokinetics, and immunogenicity (NCT05911217).

### 3.2. Allogenic Natural Killer Cell Therapy

The majority of previous attempts to sensitize PDAC to immunotherapy have been in relation to T lymphocytes; however, an area of growing interest is in innate lymphocytes or natural killer (NK) cells. NK cells are vital in immune surveillance due to their unique ability to function without the need for prior sensitization to specific antigens or the constraints of MHC [89]. Upon encountering a target (i.e., cancer or virus-infected cells), NK cells release granzymes and perforin destroying cell membranes and engaging the Fas/FasL pathway or TNF-α/TNFR-1 pathway triggering apoptosis [90]. NK cells offer several advantages over T cell-based therapies. NK cells are equipped with native activating and inhibitory receptors for tumor surveillance and antitumor activity. Unlike T cells, modified NK cells do not possess antigen specific cell surface receptors, relying primarily on recognizing the presence or absence of MHC class I molecules on target cells, thus yielding a stronger graft-versus-tumor effect. Lastly, inflammatory cytokine release is modest compared to CAR T cells, thus minimizing the risk for cytokine release syndrome (CRS) and immune effector cell-associated neurotoxicity syndrome (ICANS) [91]. To date, there have been no published clinical trials using NK cells in the adjuvant setting for PDAC. A phase I/II study is currently evaluating safety, tolerability, and preliminary efficacy of Allogeneic Magicell-NK cell infusion in patients with PDAC or cholangiocarcinoma after surgery (NCT06730009). In the phase II component, Allogeneic Magicell-NK cells will be evaluated in combination adjuvant SLOG chemotherapy (S-1, leucovorin, oxaliplatin, and gemcitabine) versus adjuvant SLOG alone. Patients will receive 6 infusions of Allogeneic Magicell-NK cells on the 11th day of each chemotherapy cycle for a total of 12 weeks of chemotherapy. Primary outcomes evaluated will be safety, dose-limiting toxicity, maximum tolerated dose, and recommended dose for the phase I component and disease-free survival for the phase II cohort.

### 3.3. Cytokine-Induced Killer (CIK) Cell Therapy

Cytokine-induced killer (CIK) cell therapy is an adoptive T-cell immunotherapy utilizing patients’ immune cells via a non-MHC-restricted pathway. Upon tumor cell recognition, CIK cells release perforin and granzyme B, creating pores in target cell membranes and triggering apoptosis in a similar mechanism to that of natural killer (NK) cells [92]. Advantages of CIK cell therapy include its ability for ex-vivo expansion, reduced alloreactivity and MHC-unrestricted tumor killing [92,93]. CIK cells given adjuvantly in hepatocellular carcinoma (HCC) have been shown to reduce recurrence and improve survival [94,95]. In a multi-center randomized controlled trial, 114 patients received CIK cells (Immuncell-LC) following potentially curative treatment for HCC. In the extended 5-year follow up, the RFS rate was 44.8% in the CIK group and 33.1% in the control group, with a HR 0.67. Notably, in the treatment arm, adjuvant CIK cell immunotherapy resulted in prolonged RFS over the 5-year period without additional boosting. An open-label, multi-center, phase 3 trial in Korea (NCT04969731) is ongoing, evaluating CIK cell therapy in combination with gemcitabine versus adjuvant single agent gemcitabine after surgical resection for PDAC. The primary outcome is recurrence free survival by independent review, with secondary endpoints of overall survival, quality of life, and CA 19–9 levels.

## 4. Future Directions in Immune-Based Strategies in the Adjuvant Setting

To date, cytotoxic chemotherapy remains the only adjuvant treatment that has consistently improved overall survival in PDAC. Despite the limitations of the existing immune-based strategies, key insights have been gained by identifying the complexities of the pancreatic TME and the interplay of immune regulation, features which must ultimately be surmounted if immunotherapy is to prove effective in PDAC. Specifically, these challenges include (1) remodeling of the TME, (2) leveraging novel therapies (e.g., direct KRAS inhibitors and next generation ICIs), and (3) optimizing patient selection and trial design.

### 4.1. Tumor Microenvironment Remodeling

Immune-based strategies, specifically cancer vaccines, have demonstrated robust and durable activation of antigen-specific T cells in response to vaccination; however, peripheral T cell activation has not reliably translated into improving disease-free or overall survival (e.g., Algenpantucel-L). A substantial challenge remains in reconciling the differences between T cell activity in the periphery and T cell infiltration at the level of the TME. T cell infiltration is one of the best predictors of response to ICI therapy and long-term survival outcomes, thus strategies to increase tumor-infiltrating lymphocytes (TILs) may provide an opportunity to improve PDAC responsiveness to ICI via upregulation of PD-1 [96,97]. In one preclinical study, intratumoral injection of the TLR9 agonist IMO-2125 in combination with systemic anti-PD-1 increased tumor-infiltrating dendritic and T cells in tumor and lymph nodes, resulting in local and distant antitumor effects in a low immunogenic murine PDAC subtype (FC1242) [98]. TIL expansion from resected PDAC specimens has been shown to be feasible, yielding TILs that are both functional and able to respond to pancreatic TAAs [99]. Efforts to investigate TIL therapy in patients with PDAC are ongoing in the advanced and metastatic setting, with two trials expanding autologous TILs from tumor resections or biopsies, followed by lymphodepleting therapy with cyclophosphamide-based regimens (NCT05098197, NCT03935893).

Future efforts to increase T cell infiltration will also need to overcome the highly desmoplastic and restrictive extracellular matrix and immunosuppressive tumor microenvironment [100]. Recently, a non-receptor cytoplasmic tyrosine kinase, focal adhesion kinase (FAK), has been shown to play an integral role in modifying stromal fibrosis and TME immune suppression [101]. In preclinical studies, the inhibition of FAK decreases the recruitment and migration of CAFs, MDSCs, TAMs, and Treg cells, ultimately leading to increased CD8+ T cell infiltration and cancer cell suppression [102,103]. In a recent phase I study, defactinib, an oral FAK inhibitor, was evaluated in combination with pembrolizumab and gemcitabine in advanced PDAC [104]. In this heavily pre-treated population, one patient (13%) had partial response and three patients (38%) had stable disease, with paired biopsy specimens demonstrating increased CD8+ T cells and reduced Tregs following treatment [104]. Defactinib, in combination with pembrolizumab, gemcitabine, and nab-paclitaxel, is currently being investigated in the neoadjuvant and adjuvant setting for PDAC (NCT03727880).

Other techniques, including radiofrequency ablation (RFA), have recently demonstrated the ability to disrupt immune evasion in PDAC in both preclinical and clinical models [105]. Mice with orthotopic syngenetic PDAC tumors who received RFA in combination with gemcitabine, anti-PD-1, and anti-CTLA-4 were found to have increased tumor antigen presentation and T cell recruitment and activation as demonstrated by whole RNA-sequencing and spatial transcriptomics [105]. On the TME level, this combination resulted in increased antigen presentation, increased immune cell infiltration of cytotoxic T lymphocytes and NK cells, and repolarization of TAMs toward an anti-tumor phenotype. This work suggests that RFA may be able to help reshape the TME towards a more pro-immune environment and increase susceptibility to subsequent ICI.

### 4.2. Leveraging Novel Agents

Another avenue for combination therapy that has not been examined thus far in adjuvant PDAC immunotherapy trials is with targeted therapy, such as direct KRAS inhibitors. Excitingly, after decades of unsuccessful attempts, direct KRAS inhibitors are now demonstrating potential to significantly impact the treatment of KRAS-driven tumors [106,107]. Early clinical trials testing these agents are demonstrating 20–30% ORR in PDAC patients, disease control rates of 90%, and unparalleled durability, with median PFS of 8 months in the pretreated PDAC setting [108,109]. Accordingly, drug development paradigms, including immunotherapy, must now consider the efficacy of novel agents in a KRAS inhibitor-exposed setting as a key component of their strategy. Fortunately, recent data have demonstrated that KRAS inhibition, both with mutation specific and multi-RAS inhibitors, remodels the TME in a manner favorable for subsequent immunotherapy approaches [110]. Multiple reports in the past year have shown that these unfavorable findings can be reversed with KRAS inhibitors, including increasing MHC I expression, decreasing MDSCs, increasing CD8+ T cell infiltration, and changing the polarity of M2 TAMs to an immunopermissive M1 phenotype [111,112]. Preclinical work has also demonstrated increased TAM sensitivity to ICI therapy, both PD-1 and CTLA-4 inhibitors. In a PDAC murine model, the KRAS G12D reversible inhibitor, MRTX1133, when combined with CXCR1/2 inhibitor and anti-LAG3 and anti-41BB antibodies yielded complete tumor regression and prolonged survival in 36% of mice at 6 months [113]. This combination revealed increased T cell infiltration and activation as well as depletion of intratumoral MDSCs and M2 TAMs [113]. Moreover, KRAS inhibitors may serve as an important tool to enable meaningful remodeling towards a pro-immune TME, enhancing sensitivity to the KRAS vaccine and ICI and translating into improved clinical outcomes.

In addition to novel antigen targets, future trials may also look to utilizing next generation ICIs. For example, unlike conventional anti-CTLA-4 inhibitors, such as ipilimumab, the novel CTLA-4 inhibitor botensilimab (AGEN-1181) has been designed with an Fc-engineered IgG1 antibody, which has been shown to enhance Fc gamma receptor (FcγR)-dependent interactions, thus promoting superior anti-tumor activity. When used in combination, botensilimab, along with balstilimab (AGEN2034), a recombinant human monoclonal IgG4κ antibody against PD-1, has been shown to have a manageable safety profile and objective clinical responses in relapsed/refractory MSS metastatic CRC [72]. Although no trials are utilizing botensilimab in the adjuvant PDAC setting, trials utilizing this combination with KRAS vaccine are ongoing in the metastatic setting (NCT06411691).

### 4.3. Optimization of Patient Selection and Trial Design

Thus far, the majority of immune-based trials in the adjuvant setting have been from phase I and phase II clinical studies with sample sizes too limited to adequately evaluate long term benefit. The evolving definition of SOC chemotherapy (e.g., Single agent gemcitabine vs. Gem/Cap vs. mFOLFIRINOX) and patient heterogeneity (e.g., Stage I–III) further adds complexity when seeking to draw conclusions from these trials. Biomarkers such as ctDNA are increasingly being evaluated in the adjuvant setting for solid tumors, including PDAC, and allow us to control for the heterogeneity in respect to determining recurrence risk and guiding decisions surrounding adjuvant therapy [114,115,116]. As ctDNA assays become validated and incorporated into the SOC protocols, it may also be a useful tool for identifying appropriate subpopulations who might benefit from immunotherapeutic approaches and measuring response to therapy. Tremendous technological advances have also been made in the tools available for tumor genomic profiling, resulting in exceedingly rapid, cost-effective, and accessible next-generation sequencing techniques [117]. In a study sequencing 336 pancreatic cancer specimens, potentially actionable targets were identified in 26% of cases, including alterations in *ERBB2*, *BRCA1* or *BRCA2*, *BRAF^V600E^*, *ROS1*, and *ALK1* [118]. Molecular profiling will therefore be crucial in designing therapies targeting either shared neoantigens (e.g., KRAS) or developing personalized neoantigen therapies.

Logistical considerations in regard to engineering and manufacturing of these various immune-based therapies will also need to be addressed prior to widespread use. For example, the various vaccine platforms possess intrinsic benefits and challenges to their manufacture and administration. Dendritic cell vaccines offer high immunogenicity and can be personalized for neoantigen-specific strategies, although production is expensive and labor-intensive. Peptide vaccines are stable and relatively simple to produce but typically require adjuvants to enhance immunogenicity. mRNA vaccines are highly versatile, do not require immune adjuvants, and have rapid scalability, but face challenges with stability and require cold chain storage [119]. Regulatory approval additionally presents with challenges unique to implementation of the various personalized therapeutics. ‘Off-the-shelf’ products with shared neoantigens appear well suited to mitigate these issues as they are individualized yet applicable to a broad population.

Sequencing of immunotherapy also remains controversial, with an increasing number of studies demonstrating the clinical benefit of neoadjuvant immunotherapy in historically ‘cold’ tumors, such as mismatch repair proficient (MMR-p) colon cancer [120]. The rationale for immunotherapy in the neoadjuvant setting may allow for more effective immune recognition of neoantigens leading to increased tumor-specific effector or memory T cells [121]. This priming effect from neoadjuvant immunotherapy has been successful in other solid tumors and there is growing interest in neoadjuvant immunotherapy for PDAC [122]. From a translational perspective, neoadjuvant trials would provide insights into how vaccination and immunotherapy remodel the TME, as resected specimens would be available to analyze. Furthermore, this approach may provide benefits not only in downstaging, thereby increasing the number of surgically resectable patients, but also may provide longer-term benefits of immunologic memory following resection.

From the strategies to date, it is clear that immune-based therapies alone will not be able to overcome the barriers presented by the PDAC TME. Sequential or combinatorial based approaches present opportunities to leverage various treatment modalities to potentiate the TME for subsequent immunotherapy. Combinatorial and sequential chemo-immunotherapy regimens have already demonstrated clear benefits in multiple tumor types, including melanoma, lung, biliary tract, and breast cancer; however, no consensus exists regarding the optimal dosing, timing, and sequence of chemo-immunotherapy [123,124,125,126]. For example, in melanoma, combination nivolumab/ipilimumab followed by BRAF and MEK inhibitor therapy is the recommended treatment sequence, whereas in early stage NSCLC, atezolizumab is indicated following adjuvant platinum-based chemotherapy [123,125]. Mechanistically, combinatorial or sequential paradigms will additionally require optimizing timing to minimize compounding toxicity and allow for lymphocyte recovery if preceded by chemotherapy or radiation. Finally, particular attention will need to be given as to how these combinatorial strategies may influence the risk for immune-related adverse events (irAEs), as the incidence of irAEs can significantly increase when ICIs are combined with chemotherapy or targeted therapies [127,128].

**Figure 2:**
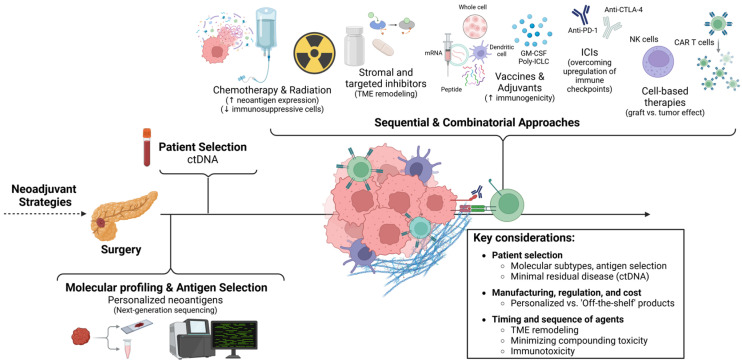
Key Considerations for Designing Adjuvant Immune-based Strategies for Pancreatic Cancer. Created in BioRender. https://BioRender.com/n72o104 (accessed on 19 March 2025).

In summary, these challenges have provided the framework on which future studies are being designed in the effort of incorporating immunotherapy in the adjuvant setting for PDAC [Figure 2].

## 5. Conclusions

With the overarching goal of eliminating residual and early micro-metastatic disease and providing durable disease control, adjuvant immunotherapy in PDAC has emerged as the most compelling solution. The adjuvant setting provides an earlier opportunity for generating immune surveillance and access to resected specimens allows for engineering of individualized personalized therapies. Thus far, the promise of adjuvant immunotherapy in PDAC remains unrealized, yet it is gaining traction rapidly with emerging data. Over the past thirty years, immune-based strategies have demonstrated their ability to generate robust tumor-specific T cells and actively remodel the TME. Results from recent trials have suggested that successful future strategies of adjuvant immunotherapy in PDAC may well require a sequential or combinatorial based approach, as the upregulation of immune checkpoints and other immunosuppressive pathways in the PDAC TME cannot be sufficiently overcome by vaccination alone. Shared antigen peptide and mRNA vaccines, in particular mKRAS vaccines, have particular promise, with the feasibility benefits of an ‘off-the-shelf’ approach, a high prevalence neoantigen target, and strong, emerging clinical data. Clinical trials involving novel combinations of chemotherapy, radiation, vaccines, ICIs, cell-based therapies, and targeted therapies are an important area of investment for our common goal of better outcomes for PDAC patients.

## Figures and Tables

**Table 1:** Adjuvant Immune-based Approaches for Pancreatic Cancer.

NCT ID/Authors	Title	Phase	Status	Treatment	Clinical and Translational Outcomes	Publications
Whole Cell Vaccines						
GVAX						
Jaffee et al., 2001	Novel Allogeneic Granulocyte-Macrophage Colony-Stimulating Factor–Secreting Tumor Vaccine for Pancreatic Cancer: A Phase I Trial of Safety and Immune Activation	I	Completed	GVAX vaccine	3/14 patients experienced delayed type hypersensitivity (DTH) responses; DFS at least 25 mo after diagnosis	[39]
NCT00389610	A Safety and Efficacy Trial of Vaccine Boosting With Lethally Irradiated Allogeneic Pancreatic Tumor Cells Transfected With the GM-CSF Gene for the Treatment of Pancreatic Adenocarcinoma	IIb	Completed	GVAX vaccine	N/A	N/A
NCT00084383	A Safety and Efficacy Trial of Lethally Irradiated Allogeneic Pancreatic Tumor Cells Transfected With the GM-CSF Gene in Combination With Adjuvant Chemoradiotherapy for Treatment of Adenocarcinoma of the Pancreas	II	Completed	GVAX vaccine, 5-FU Chemoradiotherapy	60 patients; mDFS 17.3 mo; mOS 24.8 mo. Induction of mesothelin-specific CD8+ T cells with HLA-A1+ and HLA-A2+ patients correlated with disease-free survival. Resected pancreatic cancer tissue revealed the common presence of tumor-infiltrating Tregs, which in previous PDAC studies and other solid tumors had been associated with shorter patient survival. Upregulation of PD-1/PD-L1	[40]
NCT00727441	A Randomized Three-arm Neoadjuvant and Adjuvant Feasibility and Toxicity Study of a GM-CSF Secreting Allogeneic Pancreatic Cancer Vaccine Administered Either Alone or in Combination With Either a Single Intravenous Dose or Daily Metronomic Oral Doses of Cyclophosphamide for the Treatment of Patients With Surgically Resected Adenocarcinoma of the Pancreas	II	Completed	GVAX vaccine, Cyclophosphamide	Patients who received neoadjuvant and adjuvant GVAX alone (Arm A) had trend toward longer mOS 35.0 mo vs. historical controls who received adjuvant GVAX alone 24.8 mo. 33/39 patients developed intratumoral tertiary lymphoid aggregates (TLAs). Decreased Tregs within the TLAs associated with increased intratumoral Teffector/Treg ratios and improved patient survival	[41,42]
NCT02451982	A Platform Study of Combination Immunotherapy for the Neoadjuvant and Adjuvant Treatment of Patients With Surgically Resectable Adenocarcinoma of the Pancreas	II	Recruiting	GVAX vaccine, Cyclophosphamide, Nivolumab (PD-1), Urelumab (CD137), BMS-986253 (Anti-IL-8)	Underpowered to reach statistical significance GVAX + nivolumab + urelumab (Arm C) compared to GVAX + nivolumab (Arm B) had improved mDFS (33.5 mo vs. 15.0 mo) and mOS (35.6 mo vs. 27.0 mo).	[43]
NCT01595321	Pilot Study Evaluating Allogeneic GM-CSF-Transduced Pancreatic Tumor Cell Vaccine (GVAX) and Low Dose Cyclophosphamide With Fractionated Stereotactic Body Radiation Therapy (SBRT) and FOLFIRINOX Chemotherapy in Patients With Resected Adenocarcinoma of the Pancreas	II	Completed	GVAX vaccine, Cyclophosphamide, Stereotactic Body Radiation, FOLFIRINOX	SBRT + mFOLFIRINOX + GVAX (Cohort 3) mDFS 24.1 mo and mOS 61.3 mo and which was numerically superior to overall cohort mDFS 18.2 mo and mOS 36.2 mo	[44]
Algenpantucel-L						
NCT00569387	A Phase III Study of Chemotherapy and Chemoradiotherapy With or Without Algenpantucel-L (HyperAcute^®^-Pancreas) Immunotherapy in Subjects With Surgically Resected Pancreatic Cancer	II	Completed	HyperAcute-Pancreas Immunotherapy, Gemcitabine, 5-FU Chemoradiation	1-year DFS 62%; 12-month OS 86%	[45]
**Peptide Vaccines**						
Ras/KRAS Targeted						
Gjertsen et al., 2001	Intradermal ras peptide vaccination with granulocyte-macrophage colony-stimulating factor as adjuvant: Clinical and immunological responses in patients with pancreatic adenocarcinoma	I/II	Completed	Mutant ras peptide, GM-CSF	25/43 patients (58%) peptide specific immunity was induced. Improved mOS among responders vs. non-responders (148 days vs. 61 days).	[46]
Abou-Alfa et al., 2011	Targeting Mutated K-ras in Pancreatic Adenocarcinoma Using an Adjuvant Vaccine	I	Completed	KRAS peptide vaccine, GM-CSF	24 patients resected PDAC; median RFS 8.6 mo; median OS 20.3 mo	[47]
NCT02261714	A Phase I/II Trial of TG01 and Gemcitabine as Adjuvant Therapy for Treating Patients With Resected Adenocarcinoma of the Pancreas	I/II	Completed	KRAS vaccine, Gemcitabine	4/19 patients in main cohort (vaccine during gemcitabine with serious adverse reactions. In main cohort mOS 33.1 mo and mDFS 13.9 mo. Modified cohort (no vaccine during gemcitabine) with mOS 34.3 mo and median DFS 19.5 mo.	[48]
NCT05638698	Phase II Randomized Trial Combining Tg01 Vaccine/Qs-21 Stimulon™ With Or Without Balstilimab As Maintenance Therapy Following Adjuvant Chemotherapy In Patients With Resected Pancreatic Cancer(TESLA)	II	Not yet recruiting	KRAS Vaccine, Balstilimab (PD-L1)	N/A	[49]
NCT00300950	A Phase 2 Double-Blind, Placebo Controlled, Multi-center Adjuvant Trial of the Efficacy, Immunogenicity, and Safety of GI-4000; an Inactivated Recombinant Saccharomyces Cerevisiae Expressing Mutant Ras Protein Combined With a Gemcitabine Regimen Versus a Gemcitabine Regimen With Placebo, in Patients With Post-resection R0/R1 Pancreatic Cancer With Tumor Sequence Confirmation of Ras Mutations.	II	Completed	GI-4000, Gemcitabine	Similar RFS for the GI-4000 and placebo groups 354 and 357 days, respectively (HR = 1.01 [95% CI 0.73–1.41], *p* = 0.936). Reduction in Tregs was observed in R0/R1 subjects treated with GI-4000 compared to placebo (*p* = 0.033)	[50]
NCT04853017	First in Human Phase 1 Trial of ELI-002 Immunotherapy as Treatment for Subjects With Kirsten Rat Sarcoma (KRAS) Mutated Pancreatic Ductal Adenocarcinoma and Other Solid Tumors	I	Active, not recruiting	KRAS peptide vaccine	25 patients (20 PDAC; 5 CRC). 21/25 patients with mKRAS-specific T cell responses; 21/25 patients with tumor biomarker responses; biomarker clearance 6/25; mRFS 16.33 mo	[51]
NCT05726864	First in Human Phase 1/2 Trial of ELI-002 7P Immunotherapy as Treatment for Subjects With Kirsten Rat Sarcoma (KRAS)/Neuroblastoma RAS Viral Oncogene Homolog (NRAS) Mutated Pancreatic Ductal Adenocarcinoma (PDAC) and Other Solid Tumors	I/II	Recruiting	KRAS peptide vaccine	N/A	N/A
NCT04117087	Pooled Mutant KRAS-Targeted Long Peptide Vaccine Combined With Nivolumab and Ipilimumab for Patients With Resected MMR-p Colorectal and Pancreatic Cancer	I	Active, not recruiting	KRAS peptide vaccine, Nivolumab (PD-1), Ipilimumab (CTLA-4)	8/11 patients mounted a >5–fold increase in IFNγ-producing mKRAS-specific T cells post vaccination; Improved DFS compared to non-responders (not reached vs. 2.8 mo; *p* = 0.045).	[52]
Neoantigen Peptide Vaccines						
NCT03558945	Clinical Trial to Evaluate Safety and Effect of Personalized Neoantigen Vaccine for Pancreatic Tumor Following Surgical Resection and Adjuvant Chemotherapy	Ib	Recruiting	Neoantigen peptide vaccine	3-year RFS rate 56%; 3-year OS rate 74% Expansion of cytotoxic CD8+ T cells at the priming phase with CD4+ T cells during boosting phase. Helper B-cell subtype which interacted with T cells in patients associated with prognosis	[53]
NCT04810910	Clinical Study of a Personalized Neoantigen Vaccine in Pancreatic Cancer Patients Following Surgical Resection and Adjuvant Chemotherapy	I	Recruiting	Neoantigen peptide vaccine, GM-CSF	N/A	N/A
NCT06344156	Adjuvant Therapy of Neoantigen Vaccine Plus Anti-PD-1 and Chemotherapy in Patients With Resected Pancreatic Cancer	I	Recruiting	Neoantigen peptide vaccine, Gemcitabine, Capecitabine, Tislelizumab (PD-1)	N/A	N/A
Heat Shock Protein Peptide Complex						
Maki et al., 2007	A phase I pilot study of autologous heat shock protein vaccine HSPPC-96 in patients with resected pancreatic adenocarcinoma	I	Completed	Autologous HSPPC-96 (gp96, Oncophage)	Median OS 2.2 years. Autologous-HSPPC-96 ELISpot reactivity increased significantly in 1/5 patients, no observed correlation between immune response and prognosis	[54]
**Dendritic Cell Vaccines**						
Lepisto et al., 2008	A phase I/II study of a MUC1 peptide pulsed autologous dendritic cell vaccine as adjuvant therapy in patients with resected pancreatic and biliary tumors	I/II	Completed	MUC1 peptide-loaded dendritic cell vaccine	4/12 patients alive without evidence of recurrence >4 years	[55]
Lau et al., 2022	Autologous dendritic cells pulsed with allogeneic tumour cell lysate induce tumour-reactive T-cell responses in patients with pancreatic cancer: A phase I study	I	Completed	Allogeneic tumor lysate-loaded autologous monocyte-derived dendritic cell vaccine	7/10 patients without disease recurrence of progression at median follow up of 25 months. Following vaccination peripheral blood with increased memory CD4+ T cells expressing Ki67+PD-1+ (phenotype seen in patients with improved survival following anti-PD-1 in NSCLC)	[56,57]
NCT04157127	Phase I Study of Th-1 Dendritic Cell Immunotherapy in Combination with Standard Chemotherapy for the Adjuvant Treatment of Pancreatic Adenocarcinoma (DECIST)	I	Active, not recruiting	Autologous DC vaccine	N/A	N/A
NCT04627246	A Phase Ib Study of the Combination of Personalized Autologous Dendritic Cell Vaccine and Standard Of Care Adjuvant Chemotherapy Followed by Nivolumab for Resected Pancreatic Adenocarcinoma	Ib	Recruiting	Autologous Dendritic Cell Vaccine Loaded with Personalized Peptides (PEP-DC vaccine)	N/A	[58]
NCT03592888	Pilot Study of Mature Dendritic Cell Vaccination Against Mutated KRAS in Patients With Resectable Pancreatic Cancer	I	Completed	Mature dendritic cell (mDC3/8) vaccine (primer and booster)	Median time of follow up of 25.3 months, 5 patients were without evidence of tumor recurrence. 6/9 (67%) patients generated mutant KRAS specific T cell responses	[59]
**mRNA Vaccines**						
NCT04161755	Phase 1 Clinical Trial of Personalized Neoantigen Tumor Vaccines and Programmed Death-Ligand I (PD-L1) Blockade in Patients With Surgically Resected Pancreatic Cancer	I	Active, not recruiting	mRNA neoantigen vaccine, Atezolizumab (PD-L1), mFOLFIRINOX	Responders vs. non-responders (mRFS not reached vs. 13.4 months (*p* = 0.003). In responders, vaccination expanded multiple clones (median 7.5 clones) from undetectable levels to up to 10% (median 2.8%).	[60]
NCT05968326	A Phase II, Open-Label, Multicenter, Randomized Study of the Efficacy and Safety of Adjuvant Autogene Cevumeran Plus Atezolizumab and mFOLFIRINOX Versus mFOLFIRINOX Alone in Patients With Resected Pancreatic Ductal Adenocarcinoma	II	Recruiting	mRNA neoantigen vaccine, Atezolizumab (PD-L1), mFOLFIRINOX	N/A	N/A
NCT06496373	Clinical Study of XP-004 Personalized mRNA Tumor Vaccine Combined With PD-1 Inhibitor for Postoperative Adjuvant Therapy for Pancreatic Cancer in Patients With Advanced Solid Tumors	I	Recruiting	mRNA vaccine, PD-1 inhibitor	N/A	N/A
NCT06353646	Efficacy and Safety Trial of XH001 (Neoantigen Cancer Vaccine) Sequential Combination With Ipilimumab and Chemotherapy for Patients With Resected Pancreatic Cancer	N/A	Not yet recruiting	mRNA neoantigen vaccine, Ipilimumab (CTLA-4), Gemcitabine + Capecitabine	N/A	N/A
**Adoptive Cellular Therapies**						
NCT05911217	An Open-label, Single-arm, Multicenter, Phase Ib Clinical Trial to Evaluate the Efficacy and Safety of CT041 Autologous CAR T Cell Injection After Adjuvant Chemotherapy in Subjects With Pancreatic Cancer	I	Recruiting	CT041 autologous CAR T-cell injection	N/A	N/A
NCT06730009	A Dose-Finding Phase I Followed by a Phase II Study to Evaluate the Safety and Efficacy of Allogeneic NK-cell Combined with Chemotherapy in Patients with PDA or Cholangiocarcinoma After Surgery	I/II	Recruiting	S-1, leucovorin, oxaliplatin, and gemcitabine (SLOG) + Allogeneic NK cell	N/A	N/A
NCT04969731	An Open-label, Randomized, Multi-center, Parallel, Phase III Clinical Trial to Evaluate the Efficacy and Safety of Adjuvant Immuncell-LC Therapy Combined With Gemcitabine Versus Adjuvant Gemcitabine Single Therapy After Resection in Patients With Pancreatic Ductal Adenocarcinoma	III	Recruiting	Autologous Cytokine Induced Killer Cells, Gemcitabine	N/A	N/A
Stromal-targeting Therapies						
NCT03727880	A Randomized Phase II Study of Pembrolizumab With or Without Defactinib, a Focal Adhesion Kinase Inhibitor Following Chemotherapy as a Neoadjuvant and Adjuvant Treatment for Resectable Pancreatic Ductal Adenocarcinoma (PDAC)	II	Recruiting	Pembrolizumab (PD-1), Defactinib (FAK)	N/A	N/A
